# Die globale Corona-Pandemie im Spiegel persönlicher Postings – Plattformbezogene Kommunikationsmodi in Sozialen Medien

**DOI:** 10.1007/s11614-021-00465-w

**Published:** 2021-11-30

**Authors:** Paul Eisewicht, Nico Steinmann, Pauline Kortmann

**Affiliations:** FK17 Sozialwissenschaften, TU Dortmund, Emil-Figge-Str. 50, 44227 Dortmund, Deutschland

**Keywords:** COVID-19, Corona, Soziale Medien, Imgur, Memes, Multimodalität, Grounded Theory, Filter Loops, Plattform Vernacular, COVID-19, Corona, Social Media, Imgur, Memes, Multimodality, Grounded Theory, Filter Loops, Platform Vernacular

## Abstract

Der Beitrag typisiert anhand der Plattform Imgur.com und der dortigen User*innenbeiträge individuelle Erfahrungen wie auch kollektive Verhandlungen der Krisenhaftigkeit und des Umgangs damit im Zuge der COVID-19 Pandemie. Hierfür wird auf ein Sample aus 2 % der am besten bewerteten Beiträge (645 Postings), die unter dem Hashtag #coronavirus veröffentlicht wurden, zurückgegriffen. Die Beiträge wurden nach den Prinzipien der (Visuellen) Grounded Theory kodiert und angelehnt an eine Mixed-Method-Grounded-Theory deskriptiv-statistisch ausgewertet. Dabei ist nicht nur auffällig, welche Vielfalt an Medienformaten genutzt wird, sondern auch, dass hier eigene Beiträge neben weiterverwendeten Beiträgen von anderen Sozialen Medien stehen. Die Plattform selber ist damit auch ein Filter der Beiträge von anderen Plattformen. Zudem stehen persönliche Beiträge zum Umgang mit der Pandemie neben Verhandlungen politischer Kritik und informativen Postings. Entlang des ausgewerteten Datenmaterials lässt sich so zeigen, dass User*innen verschiedene Kommunikationszwecke, etwa den der Unterhaltung, der Information und des sozialen Zusammenschlusses, in schneller Abfolge auf derselben Plattform miteinander verbinden können. Ein Schwerpunkt der Analysen liegt auf dem Medienformat der Memes, die in sozialen Medien eine prominente Rolle einnehmen und die aufgrund ihrer Multimodalität und ihrem auf adaptiver Serialität beruhenden Verweisungszusammenhang Herausforderungen an den Prozess der Datenerhebung und -auswertung stellen, deren Reflexion anhand des vorliegenden Materials neue Impulse für die Erforschung von Memes und der Kommunikation auf Sozialen Medien liefert.

## Abstract (en)

The article typifies individual experiences as well as collective negotiations of the crisis and the handling of it in the course of the COVID-19 pandemic on the basis of the platform Imgur.com. For this, we draw on a sample of 2% of the highest rated posts (645 posts) published under the hashtag #coronavirus. The posts were coded according to the principles of (Visual) Grounded Theory and descriptively-statistically analyzed following a mixed-method approach. It is not only noticeable how many different media formats are used, but also that the participants’ own posts appear alongside posts from other social media that have been reused. The platform itself is thus also a filter for contributions from other platforms. In addition, personal posts on dealing with the pandemic appear alongside political criticism and informative postings. Along the analyzed data material, it can thus be shown that users can combine different communication purposes, such as entertainment, information and social connection, in rapid succession on the same platform. One focus of the analyses is on the media format of memes, which play a prominent role in social media and which, due to their multimodality and their reference context based on adaptive seriality, pose challenges to the process of data collection and analysis. The reflection on the basis of the material therefore provides new impulses for the study of memes and communication on social media.

## Globale Krisen im Spiegel persönlicher Erfahrungen und soziale Medien als Forschungsgelegenheit

Globale Krisen, wie die Covid-19-Pandemie, fordern die Soziologie heraus, wie globalpandemische makroökonomische und nationalpolitische Veränderungen beschrieben und analysiert werden können (Lessenich 2020; Scambler 2020). Sie werfen aber auch die Frage auf, wie diese Krisen im Alltag wahrgenommen werden. Die Frage nach der Außergewöhnlichkeit der Krise auf gesellschaftlicher muss um die auf individueller Ebene komplementiert werden (Katila et al. 2020). Auf Ebene individueller Erfahrungen gilt es, die Erfahrung der Krisenhaftigkeit der Pandemie, Veränderungen in der Lebensführung und Reflexionen der Selbstpositionierung zu rekonstruieren. Mutmaßlich stellt die anhaltende globale Pandemie und das sich ständig wandelnde (Un‑)Wissen um den Erreger, seine gesundheitlichen Auswirkungen und die Wirksamkeit von Präventions- und Schutzmaßnahmen eine große Herausforderung für Menschen dar. Entgegen einer Post-Corona-Gesellschaft scheint sich hier die zweite Moderne in ihrer individualisierten Risikohaftigkeit (Beck 1986) und der Reflexivität der – über verschiedene interdependente Expertensysteme vermittelten – Wissensbestände (vgl. Giddens 1996) deutlichzumachen.

In diesem Beitrag fokussieren wir auf Inhalte Sozialer Medien, um einen Zugriff auf die Darstellung individueller Erfahrungen (durch Posts) wie auch kollektive Verhandlungen der Pandemie (über Kommentare und Likes) zu bekommen. Die Entwicklung und Verbreitung digitaler Infrastrukturen und Plattformen (Nieborg und Poell 2018) wie auch die Aneignung durch User*innen, die über Soziale Medien kommunizieren, ihr Handeln dokumentieren und sich in der Beobachtung ihrer sowie der Darstellungen anderer verorten (vgl. Jones 2015), kennzeichnet eine forschungsbegünstigende Besonderheit. Die zunehmend institutionalisierte Selbst-Veräußerung im digitalen Panoptikum (vgl. Hitzler 2014) ermöglicht einen Zugriff auf Daten, die aber hinsichtlich ihrer medientechnischen Vermittelbarkeit reflektiert werden müssen. Als prominente Medienformate in Sozialen Medien werden dabei Memes verhandelt (Grundlingh 2018). Der Begriff des ‚Meme‘ geht auf den Biologen Richard Dawkins zurück, der diesen zur Beschreibung der Entwicklung von Kulturen in Form ideeller, replikatorischer Einheiten als ein Pendant zum Genbegriff einführte. Unter einem Internet-Meme versteht man „a unit of information (idea, concept or belief), which replicates by passing on via internet […] in the shape of a hyper-link, video, image, or phrase. It can be passed on as an exact copy or can change and evolve“ (Díaz 2013, S. 97). Shifman (2014) definiert Internet-Memes in ähnlicher Weise, plädiert jedoch dafür, diese als Gruppen von (digitalen) Daten zu fassen, die „common charateristics of content, form, and/or stance“ aufweisen, miteinander in explizitem Bezug stehen und mehrfach im Internet genutzt und verändert genutzt werden (ebd., S. 41). Prototypisch kennzeichnet Internet-Memes eine Bild-Text-Relation (vgl. Molina 2020). Memes sind in die Kultur derjenigen eingebettet die sie nutzen, und können entsprechend unterschiedlich gelesen und verstanden werden (ebd., S. 384; Brown 2013, S. 190 f.). Im weitesten Sinne sind sie semiotische Ressourcen, deren Bedeutung sich aus einem Verweisungssystem erschließt, welches von sozialen Gruppen angewandt und reproduziert wird (Grundlingh 2018, S. 4 f.). Die Popularität von Memes liegt dabei (a) in der einfachen Nutzung bereitgestellter Vorlagen und kostenfrei nutzbarer Plattformen zur Produktion und Distribution von Mitteilungen (Rintel 2013), die (b) informativen Gehalt mit einer affektuellen Komponente verknüpfen (Heath et al. 2001) und (c) darin verschiedene Kommunikationsfunktionen bedienen. Die Diskussion um Falschnachrichten und Verschwörungstheorien in Sozialen Medien (zu Corona Boberg et al. 2020; Bruns et al. 2020) zeugt von deren gestiegener Bedeutung als Informationsquelle zur Meinungsbildung (zu Corona auf Twitter Wicke und Bolognesi 2020; Vicari und Murru 2020).

Dementsprechend finden sich zahlreiche Arbeiten zu Memes im Zuge der Corona-Pandemie (Dynel 2020; Hussein und Aljamili 2020; Pauliks 2020; Pulos 2020), welche diese als Zugang zu persönlichen Erfahrungen und Emotionen (Cipolletta und Ortu 2020) und zu politischen Diskursen (Saint Laurent et al. 2021) nutzen. Zur Eingrenzung finden sich dabei plattformspezifische Analysen (Wicke und Bolognesi 2020; Boberg et al. 2020) wie auch Analysen mit nationalstaatlichem Fokus (Flecha Ortiz et al. 2020; Oduor and Kodak 2020). Melissa Meyer (2021) argumentiert, dass sich Memes nicht nur als Zugang zum Verständnis der Pandemie eignen, da sie selber bedeutungsgenerierend und -stabilisierend sind, sondern auch, dass sich Memes im Zuge der Pandemie selber transformieren (aus journalistischer Sicht Romano 2020) (Tab. 1).

**Table 1:** 

Autor*Innen	Plattform(en)	Zeitraum	Datenerhebung	Auswertung
Dynel (2020)	9gag, Reddit, Imgur, Twitter	Jan. 2020–April 2020	Suchbegriffe: ‚#COVID19‘, ‚#coronavirus‘, ‚meme‘ und ‚face mask‘; getaggt als: ‚funny‘ oder ‚humo(u)r‘; min. drei Nutzerreaktionen	Grounded Theory und multimodale Diskursanalyse
Flecha Ortiz et al. (2020)	Interviews über Memes	März 2020	Online Survey	Quantitativ (PLS-SEM)
Pulos (2020)	U. a. cheezburger.com, me.me, Reddit, Imgflip	keine Angabe	Erhebung via Google Images; Fokussierung auf Meme-Typen ‚Macro‘, ‚shop‘ oder einen Hybrid; Suchbegriffe inspiriert durch ‚COVID-19 Vocabulary‘ von Kathy Katella (2020)	Exemplarische Auswahl
Oduor und Kodak (2020)	Fünf Whatsapp-Gruppenchats (921 Teilnehmende)	13. März 2020–1. April 2020	25 Memes; jeweils mit min. fünf Antworten; Fokus: Nutzung von Humor	Kodierende Textanalyse/Computer Mediated Discourses Analysis
Saint Laurent et al. (2021)	Reddit	23. Jan. 2020–17. Mai 2020	Subreddit ‚r/CoronavirusMemes‘; 273 Memes betrachtet; Fokus auf politische Bezüge	Quantitatives Kodieren; anschließend Inhaltsanalyse
Hussein and Aljamili (2020)	Jordanische Seiten auf Instagram und Facebook	März 2020–Mai 2020	Auswahl von 50 Memes und Karikaturen basiert auf ‚Likes‘; Fokus auf Humor	Social Semiotic Approach

Neben der Frage, wie die Pandemie in den Sozialen Medien verhandelt wird, geht es uns auch darum, welchen Beitrag eine Pandemieforschung zum mediensoziologischen Verständnis von Memes leisten kann. Dabei fällt in der Forschungsliteratur auf, dass die Begrifflichkeit von Memes noch strittig ist bzw. Memes oft auf eine Funktion reduziert werden (vgl. aber Grundlingh 2018). Dies führt dann zu entsprechend fokussierten Fallstudien mit entsprechenden Samplingproblemen. Daher behandelt der Beitrag auch, wie eine begrifflich-konzeptionell adäquate und methodisch angemessene Forschung im Kontext digitalisierter Kulturproduktion (vgl. Nieborg und Poell 2018) gestaltet sein kann.

Bevor die empirischen Daten entlang der medienformatbezogenen Einbettung der Beiträge, der sie kennzeichnenden kommunikativen Muster und der in ihnen verhandelten Themen dargestellt werden, widmet sich das folgende Kapitel zentralen Herausforderungen der Memeforschung und expliziert in einem zweiten Schritt die dem vorliegenden Artikel zugrundeliegende methodische Anlage.

## Memes in Sozialen Medien: Herausforderungen und methodische Anlage

### Herausforderungen der Memeforschung

Der Zugang über User*inneninhalte auf Sozialen Medien zur individuellen Bedeutungszuschreibung angesichts der Pandemie ist insofern limitiert, als es sich um einen medientechnisch vermittelten Zugang zur Lebenswelt der Handelnden handelt, der dahingehend zu reflektieren ist (zur Forschung unter Mediatisierungsbedingungen Eisewicht 2015, S. 99 ff.). In der Literatur werden hinsichtlich der Analyse digitaler und nutzer*innengenerierter Daten (Dynel 2020, S. 179; Molina 2020) eine Vielzahl an methodischen Zugängen diskutiert. Teils werden gängige Methoden auf die ‚digitalen Artefakte‘ (Wiggins und Bowers 2015; als Medienartefakte Grundlingh 2018) übertragen (vgl. etwa Moebius 2018), teils werden neue methodische Zugänge entwickelt (Pauliks und Ruchatz 2020). Auch in der Erhebung unterscheiden sich die Ansätze: von der Analyse einzelner Medieninhalte, der vergleichenden Analyse verschiedener Fälle (Gibbs et al. 2014), der Analyse von Medieninhalt und Kommentierung, der Einbettung des Inhalts in das Profil des/der Sprecher*in und/oder in den Kontext der Website, bis hin zur Analyse von Codes und Algorithmen, welche der digitale Stoff sind (Grenz und Eisewicht 2020), aus dem sich am Bildschirm das analoge Bild materialisiert (Pauliks und Ruchatz 2020). Mit Fokus auf Internet-Memes, als typischen Medieninhalt, lassen sich folgende methodisch zu berücksichtigende Eigenheiten ausmachen:

1. Es handelt sich bei Memes um die Adaption einer bereitgestellten *Vorlage* (als Templatability Rintel 2013). Wenn sie als Memes verbreitet werden, haben diese eine (Entstehungs‑)Geschichte hinter sich, im Rahmen derer sich ästhetisch-sinnhafte Konventionen etablieren. Dabei wird vorliegendes Ausgangsmaterial aus der Popkultur (aus Filmen, Musikvideos, Comics u. Ä.) oder Bildmaterial zum Weltgeschehen (Sport, Politik etc.) adaptiert – oder aber es werden eigens angefertigte Zeichnungen erstellt (die Beispiele bei Pauliks und Ruchatz 2020).
2. Zweitens handelt es sich um *multimodale* Daten (Pulos 2020; Dynel 2020), d. h. ein Meme ist mehr als nur seine bildliche Ebene, mindestens eine Text-Bild-Relation (Bülow und Johann 2018; Molina 2020), aber auch als Bewegtbild mit Ton oder (animiertem) Text möglich. Dies wirft die Frage danach auf, wie die verschiedenen Medienmodi sinnhaft verschränkt sind.
3. Die Möglichkeit der Nutzung von Vorlagen führt zur schnellen Verbreitung, aber auch zu Anpassungen und Modifikationen. Memes zeichnet folglich eine adaptive *Serialität* (Pauliks 2017; als *echoing* Dynel 2020) aus. Nicht nur stehen verschiedene mediale Ebenen in Relation, das Meme selbst steht auch in Relation zu seiner Vorlage, zu anderen Anwendungen und zu anderen Memes.
4. In ihrer Serialität und der Adaption auf verschiedene Situationen stehen Memes in einem *Verweisungszusammenhang*. Die Bedeutung, die Memes auf einzelnen Plattformen und über diese hinweg zugeschrieben wird, speist sich nicht nur aus dem internen Verweisungszusammenhang (seiner Serialität) und der Konstellation zu anderen Memes, sondern auch aus dem soziohistorischen Kontext, in dem das Meme verwendet wird. Und es adressiert seitens der/des Sprechers*in ein entsprechend lesefähiges Publikum, das diese Verweise versteht (Grundlingh 2018, S. 5 f., 12 f.).

In Anknüpfung an diese Ausführungen bedarf es auch bei Forschenden einer Literalität bezüglich dieser multimodalen, plattformübergreifenden und -internen Verweisungszusammenhänge, die den Boden für die gruppenspezifischen Bedeutungshorizonte des Memes bereiten.

**Figure 1:**
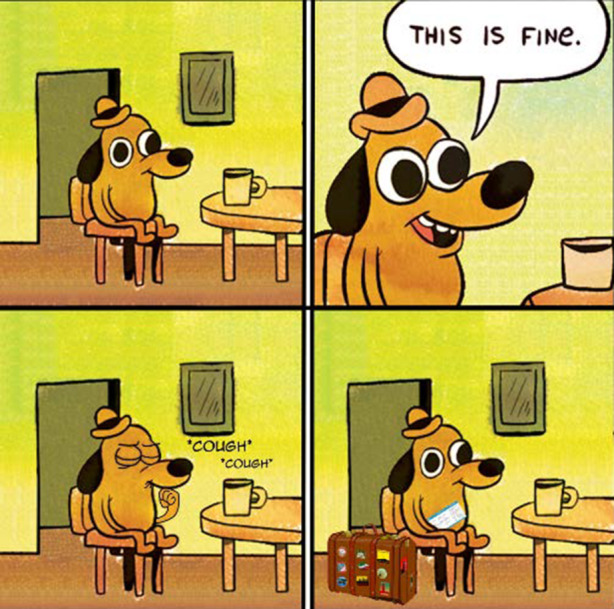

**Figure 2:**
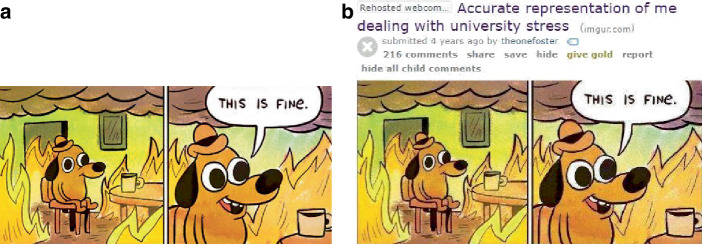

**Figure 3:**
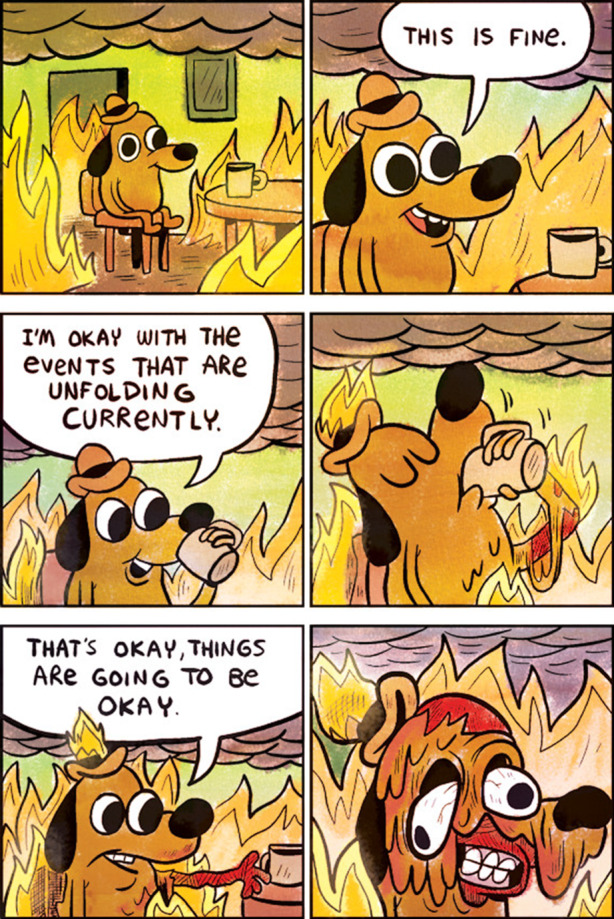

In Abb. 1 sieht man z. B. einen im Comicstil gezeichneten, anthropomorphisierten Hund mit Hut in einem Zimmer auf einem Stuhl an einem Tisch mit einer Tasse. Der Hund sitzt im ersten Bild auf dem Stuhl, das zweite Bild zeigt eine Nahaufnahme und eine Sprechblase, in der der Hund ausdrückt, dass „das fein/gut ist“. Im dritten Bild sieht man ihn husten und im vierten Bild mit einem Koffer neben dem Stuhl und einem Flugticket unter dem Arm. Was mit dieser Bild-Text-Folge gemeint ist, wird deutlich, wenn man die Vorlage des Memes und andere Anwendungen hinzuzieht (Abb. 2). Es fällt auf, dass aus der Vorlage das Feuer, in dem der Hund sitzt, entfernt wurde, und dass das Meme auf Situationen anspielt, in denen eine Fehlwahrnehmung der Bedrohungslage (des Feuers) seitens des Hundes vorliegt (z. B. in einer frühen Anwendung den Umgang mit Universitätsstress kommentierend; Abb. 2b). Hier liegt eine Kohärenz zum Originalmaterial vor (Abb. 3), das die Thematik der (Selbst‑)Verleumdung ausführt. In Bezug auf das Bild aus unserem Sample wird erkenntlich, dass die im Hund repräsentierte Person sich der durch die Coronapandemie verursachten Gefahrenlage nicht im Klaren zu sein scheint, vielmehr, dass sie selber womöglich infiziert ist (im Husten angezeigt) und sich nicht von Flugreisen abhalten lässt – und damit zur Verbreitung des Erregers beiträgt. Im Unterschied zu anderen Anwendungsfällen der Vorlage scheint hier eine Differenz zwischen den im Hund repräsentierten Personen und dem/der Ersteller*in des Memes markiert, indem das pandemieleugnende Verhalten durch die Flugreiseanzeichen, in Analogie zu dem Feuer der Originalvorlage, dramatisiert wird.

Typischerweise sind Memes in persönliche Postings und/oder auf den Profilseiten von User*innen eingebettet und je nach Plattformmöglichkeiten und eigenen Einstellungen für ein (un)bestimmtes Publikum einsehbar. Mit der Einbettung in eine Website bzw. Plattform ergeben sich weitere Besonderheiten, die nicht nur für Memes, sondern insgesamt für (persönliche) Postings gelten:

- Bei vielen Plattformen besteht ein *Hierarchieproblem* hinsichtlich der Einordnung der Postings. Zwar lassen sich auf den meisten Seiten Kennzahlen der Popularität finden (als Likes, Upvotes u. Ä.), diese sind jedoch dadurch determiniert, wer Beiträge sehen kann, wie viele Follower/Freunde der/die Postende hat usw. Damit ergibt sich das Problem, dass weder für die Plattform noch für die User*innengruppe die Relevanz der Beiträge klar rekonstruiert werden kann. Viele Arbeiten umgehen dieses Problem durch exemplarische Fallanalysen (die jedoch willkürlich wirken). [Hierarchie- als *Auswahlproblem*]
- Ein weiteres Problem besteht in der *Dynamik* der Plattformen und der *Flüchtigkeit* der Postings (Schirmer et al. 2015). Beiträge werden gelöscht oder verändert, Likes werden hinzugefügt oder abgezogen. Erstellte Samples veralten bei zeitaktuellen Ereignissen innerhalb weniger Wochen und werfen Fragen nach der Archivierung auf. In welchem Umfang sollen Postings gesichert und so aufbereitet werden, dass sie gegebenenfalls mit entsprechender Analysesoftware weiterverarbeitet werden können? Websites lassen sich als ganze Seite speichern, als Screenshot usw., womit aber eine Reduktion des Datums einhergeht [Flüchtigkeits- als *Datenaufbereitungsproblem*].
- Letztlich besteht auch eine Unsicherheit hinsichtlich der Sprecher*innenposition. Oft finden sich außer einem User*innennamen nur wenige Informationen zum/zur Postenden. So kann es sich z. B. auch um sogenannte Bots handeln, die posten, liken etc. und so ein ganz eigenes selbstreferentielles technisches Kommunikationssystem bilden. [Anonymisierungs- als *Identifikationsproblem*]

**Figure 4:**
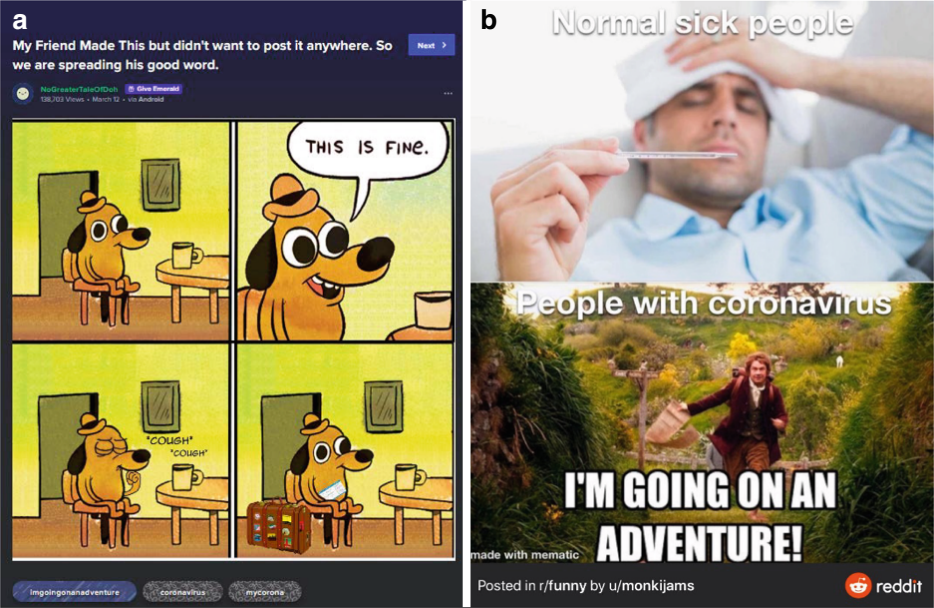

**Figure 5:**
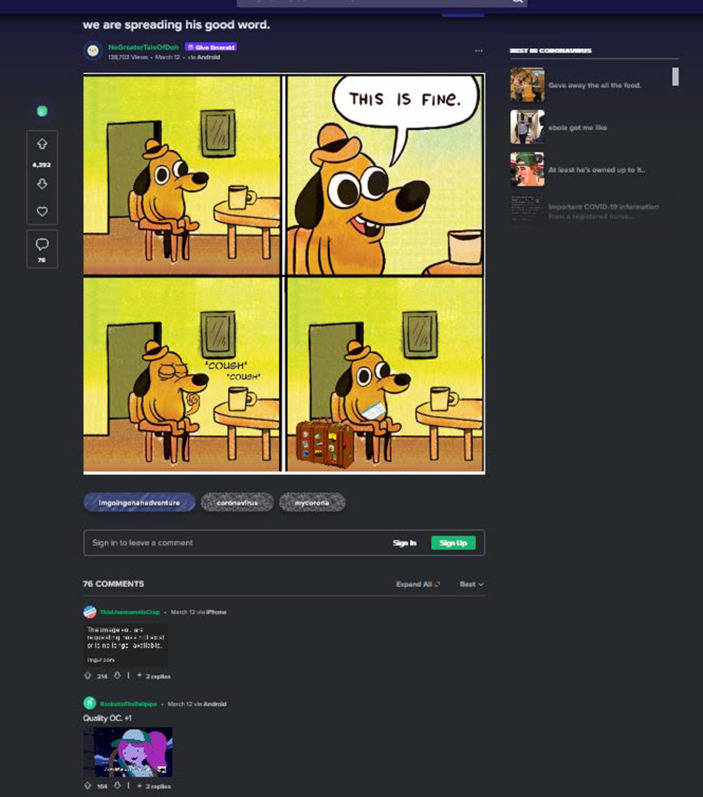

**Figure 6:**
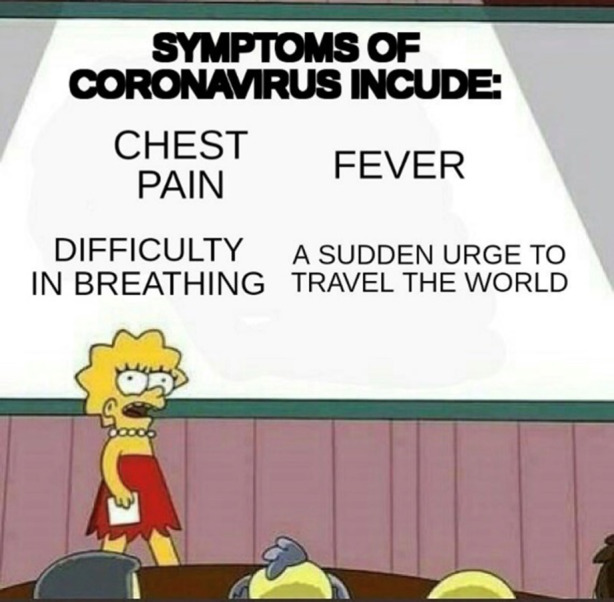

Aus den Eigenheiten der Plattform ergeben sich Fragen nach der Auswahl der Erhebungseinheiten und der Auswahl der Einheit für sich. Dies wird in den Abb. 4, 5 und 6 deutlich. In Abb. 4a ist das Meme im Kontext des Postings ersichtlich. Oben kommen ein Titel, ein User*innenname und unten eine Reihe an Hashtags hinzu. So z. B. „Coronavirus“ und „Mycorona“ als Einbettung in die Pandemie, aber auch „imgoingonanadventure“ als Verweis auf ein Meme aus dem Film der Hobbit (als Verweis auf die Sorglosigkeit der mit dem Hund repräsentierten Menschen; vgl. Abb. 4b für vergleichbares Coronameme). Hinzu kommen Metadaten der Plattform, wie das Datum des Postings, das benutzte Endgerät und die Zahl der Ansichten. Eine weitere Möglichkeit des Ausschnitts findet sich in Abb. 5, in dem die Einbettung in die Website deutlicher wird. Rechts eine Liste mit verwandten Posts und links eine Darstellung zu den ‚Likes‘ (in diesem Falle das Verhältnis von Zustimmung und Ablehnung), die Zahl von User*innen, die den Post gespeichert oder kommentiert haben. Es folgen unten die Kommentare, die wiederum kommentiert und bewertet werden können. Auch in den sogenannten Talkbacks zum zentralen Inhalt (dem Posting) werden Bedeutungen und Zustimmung zum Posting verhandelt (im zweiten Kommentar als „quality OC“, also hochwertiger Eigenbeitrag bzw. ‚Original Content‘, gelobt). So finden sich auch in den Kommentaren Wiederholungen der Aussage des Postings, z. B. in einem weiteren Meme in der Beschreibung eines Coronasymptoms als ‚plötzlicher Drang zum Reisen‘ (vgl. Abb. 6). Über die Verweise zur Vorlage des Memes (vgl. Abb. 2), in den Hashtags (vgl. Abb. 4) und in den Kommentaren (vgl. Abb. 6), lässt sich so eine vorläufige (Be‑)Deutung rekonstruieren. Vor allem kommt hier eine Form der Kritik an persönlichem Fehlverhalten zum Ausdruck, die ein (Kern‑)Thema im Rahmen unseres Materials darstellt (dazu Abschn. 3). Weiter scheint hier ein kommunikativer Gruppenzusammenhang über die geteilte Meinung zu einer Gegengruppe zum Ausdruck zu kommen (auch deutlich in einem Kommentar zum Posting, es handle sich bei den im Hund repräsentierten Menschen um „COVIDiot“en also Idioten im Kontext der COVID-19-Pandemie).

Aus den medien- und plattformspezifischen Eigenheiten der Verwendung und Einbettung von Memes folgen u. a. Fragen der Fallauswahl, der Zusammenstellung des Samples und der Aufbereitung, die unseren methodischen Zugriff anleiten.

### Methodische Anlage

Die Problematik, wie relevante Fälle identifiziert werden können, war leitend für unsere Fallauswahl. Plattformseiten wie Facebook, Instagram und Twitter machen es aufgrund der disparaten Publikumsmengen schwierig, über die Plattform hinweg anerkannte Mitteilungen zu identifizieren oder bei Postings deren relative Popularität bzw. Relevanz zu rekonstruieren. Daher nahmen wir die Social-Media-Plattform Imgur.com in den Blick, die laut Alexa-Ranking (2021) zu den 100 meistbesuchten Seiten weltweit gehört. Die Seite bietet einen Strukturvorteil, der darin besteht, dass die Startseite (die „frontpage“) allen Nutzer*innen eine allgemeine, nicht personalisierte Anzeige populärer Postings (sogenannte „most viral“) anzeigt. D. h., die Anzeige der Posts ist kein personalisierter Feed von Beiträgen gefolgter Personen/Seiten und damit keine individualisierte und darin fragmentierte Abbildung verschiedenster Beiträge. Beiträge werden auf Imgur prinzipiell allen angezeigt (neue Beiträge in der Rubrik „user submitted“) und können von allen (angemeldeten) User*innen bewertet werden.^1^ Somit ist es möglich, sich zu einem Hashtag alle Beiträge anzeigen zu lassen und nach den Bewertungen zu sortieren, um einen Überblick über die – auf der Seite unter dem Hashtag – populären Beiträge zu erhalten.

Der mit Abstand meistgenutzte Tag zur Zeit der Erhebung (Juni 2020) war #coronavirus mit 32.250 Postings.^2^ In einem ersten Zugriff haben wir ein Sample erstellt, indem wir die Beiträge zu diesem Tag nach Popularität haben sortieren lassen und die ersten 645 Ränge in unsere Analyse einbezogen haben. Dadurch entstand zu diesem Zeitpunkt ein Datenkorpus aus 2 % der meist- und am besten bewerteten Beiträge zum Tag #coronavirus. Die Überlegung war, damit zwei Fragen nachzugehen: Welche Themen werden in Sozialen Medien generell verhandelt (ohne eine Einengung auf – politische oder humoristische – Memes) und welche Rolle spielen Memes im Gesamtkontext der Postings. Mit diesem Zugriff scheint es möglich, auf plattformbezogene geteilte Aussagen zuzugreifen und damit den konjunktiven Erfahrungsraum der mit der Pandemie befassten User*innen näherungsweise rekonstruieren zu können.^3^

In einem zweiten Zugriff wurden die Postings offen, nach den Prinzipien der Grounded Theory (Strauss und Corbin 1990) und spezieller mit Blick auf die Visuelle Grounded Theory (nach Dietrich 2020; Mey und Dietrich 2016), kodiert. Dadurch wurden aus dem Material induktiv Kodegruppen zu Themen der Postings, Informationen zu den Autor*innen und Adressat*innen, zum *Medienformat* und zum kommunikativen Muster und zu eventuell angegebenen *Quellen *entwickelt. Es ging nicht um die Anwendung eines vorgegebenen Kodierrasters, sondern um eine aus dem Datenmaterial rekonstruierte Ordnung anhand zentraler Differenzmerkmale. Hierbei wurde auf das Posting und den Titel der Beiträge fokussiert. Diese zentralen Differenzierungen sind anleitend dafür gewesen, einerseits anhand der Differenzlinien prototypische Fälle für eine detailliertere Analyse auszuwählen (hier wurden Hashtags, Kommentare und die Einbettung des Postings hinzugezogen; vgl. Abschn. 2.1) und andererseits eine deskriptive Statistik zur Verteilung der Ausprägungen der Kategorien im Sample zu erstellen. Es handelt sich um einen methodenpluralen Ansatz, wie für multimodale Phänomene gefordert (Wildfeuer et al. 2020), angelehnt an Überlegungen zu einer Mixed-Methods-Grounded-Theory (Johnson und Walsh 2019). Wir hoffen, so Aussagen über die prototypischen Themen, die medialen und kommunikativen Vermittlungsformen treffen wie auch diese mit Blick auf das Sample einordnen zu können.

## Die Pandemie im Spiegel sozialer Medienbeiträge

Im Rahmen dieses Beitrags gehen wir zwei Fragenkomplexen – der Verhandlung der Pandemie durch die User*innen und der Rolle von Memes als Teil eines partiellen visuellen Diskurses (Saint Laurent et al. 2021) nach. Hierfür soll das Sample hinsichtlich der Medienformate, der kommunikativen Muster und der verhandelten Themen vorgestellt werden.

### Medienformateinbettung – Postings zwischen Originalbeitrag und Weiterverwertung

**Figure 7:**
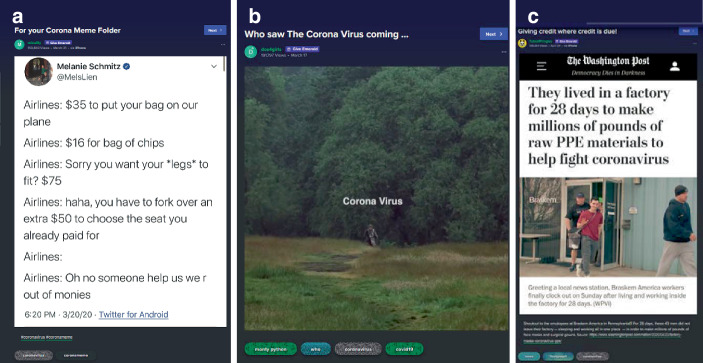

Mit Blick auf das Sample fällt auf, dass eine Breite an Medienformaten zu finden ist – Texte, Bilder, Videos mit und ohne Ton etc., die verschiedenartig in die Beiträge eingebettet sind. Bei dieser Einbettung zeigen sich drei Varianten (Abb. 7), die Einbettung als Medienbeitrag (z. B. als Screenshot eines Zeitschriftenartikels; Abb. 7c) ist dabei am seltensten (9,9 % des Samples).^4^ Häufiger finden sich Beiträge von anderen Sozialen Medien (39,4 %), v. a. Twitter (34,1 %). Die Plattform dient im Falle der Medienbeiträge und der Beiträge von anderen Plattformen als ein Filter von für die Nutzer*innen relevanten Beiträgen (als soziotechnische Rückkopplungsschleife, Davies 2018).^5^ Dabei werden diese teils durch Titel oder eigene Kommentierungen begleitet, wodurch eine ganze Reihe hybrider Formate entstehen (z. B. Zeitungsbeitrag mit Memekommentar oder als Tweet mit eigener Kommentierung). Im Falle der Screenshots von anderen Sozialen Medien sind teils Kommentare auf der Ursprungsplattform inkludiert (s. unten Abb. 13a, ‚this is the single funniest coronavirus meme‘), welche eine Bewertung des Inhalts mitliefern (und damit die Auswahl des Beitrags zur Weiterverteilung rechtfertigen).

Den anderen Teil machen uneingebettete, plattformeigene Beiträge aus (48,2 %; 2,5 % Sonstige/uneindeutig, z. B. der Screenshot eines privaten SMS-Austauschs), also Bilder, Illustrationen, Texte, Memes, Videos die z. B. Originalinhalte oder verschleierte Weiterverwendungen umfassen. Originalbeiträge können eigens erstellt sein, aber auch auf bestehendes Material zurückgreifen, z. B. Memevorlagen neu anwenden (vgl. Abb. 1) oder aber Inhalte anderer Medien (v. a. Filme und TV-Serien; Abb. 7b als Filmausschnitt aus Monty Pythons, Die Ritter der Kokosnuß; als Remediation Bolter und Grusin 2000).

**Figure 8:**
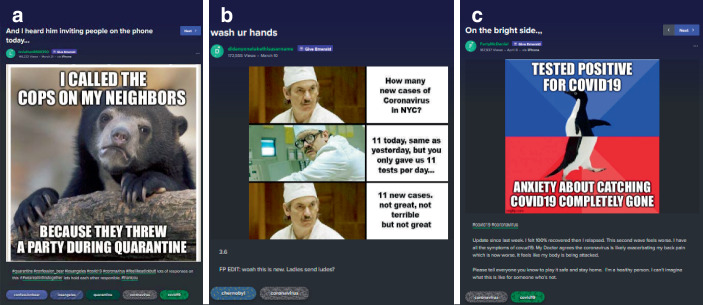

Herkömmliche Memes (vgl. Abb. 8; Bild-Text-Relationen als *standard image macro*; Grundlingh 2018, S. 9 f.) machen nur einen Bruchteil der erstellten Beiträge aus (9,5 % vom Sample), finden sich aber oft als Kommentar zum Beitrag (vgl. Abb. 6) oder als Begleitung anderer Formate. Die Plattform lässt sich schwer auf ein Medienformat reduzieren, so finden sich die behandelten Themen in den verschiedenen medialen Formaten wieder.

### Kommunikative Muster – zwischen Unterhaltung, Meinungsbildung und Information

**Figure 9:**
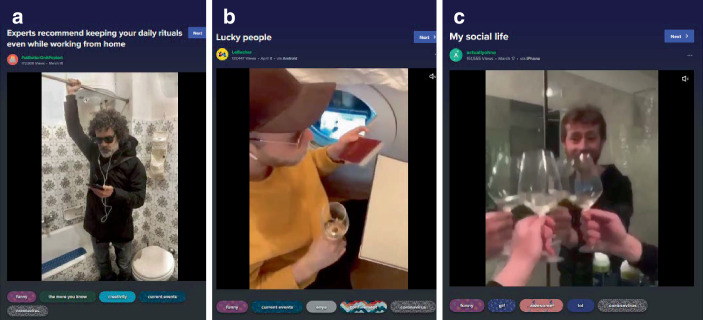

Hinsichtlich der kommunikativen Muster lassen sich drei Cluster identifizieren. Zum einen gibt es – primär unterhaltsame (komödiantische/kreative Posts; 24,7 % des Samples), welche vor allem die Folgen der Kontakteinschränkungen, häuslicher Isolation und des Homeoffice behandeln (Abb. 9). Hier ist ein exemplarischer Untertypus abgebildet: Zu sehen sind Einzelpersonen im Haushalt (bei der Badewanne, der Waschmaschine und einem Spiegel), die eine Handlung zeigen (sich an einer Haltestange in der Bahn festhalten, Flugzeug fliegen, auf einem Treffen miteinander anstoßen), die typischerweise nicht in diesen Räumen und zudem in Anwesenheit anderer Personen stattfindet. Dadurch, dass diese alltäglichen, öffentlichen, geselligen Handlungen in isolierte Privaträume des Haushalts verlegt werden, wird so der Effekt der Vereinzelung und des Verzichts auf diese Tätigkeiten humoristisch als Bruch mit Routinen und Alltagserfahrungen markiert. Damit werden hier z. B. auch thematisch Herausforderungen des Umgangs mit den Maßnahmen erkennbar (ohne explizite Bewertung dieser, außer, dass den vorgegebenen Regeln gefolgt wird).

**Figure 10:**
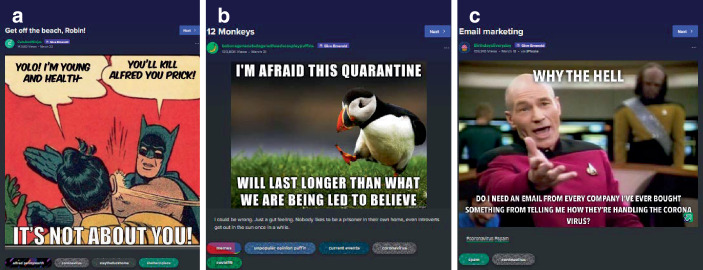

Es gibt auch Beiträge, die expliziter eine Meinung zum Umgang mit der Pandemie zum Thema haben (22,6 % des Samples). Hier lassen sich viele Memes finden, die Kritik zum Ausdruck bringen (Abb. 10; auch Abb. 2 und 8b; im Kontrast z. B. zur persönlichen Erzählung qua Meme in Abb. 8c). Hier findet sich vorrangig die Unterstützung der Schutzmaßnahmen und Kritik an Politik, Unternehmen und Einzelpersonen, welche die Pandemie nicht ernst nehmen oder zu ihrem Vorteil nutzen.

So ist in dem Batman-Slapping-Robin-Meme (Abb. 10a) ein Comicbild von Batman und Robin zu sehen, in der Robin mit dem Rücken zum Betrachter – als Repräsentant Jugendlicher seine Gesundheit und Jugend markiert (auch in der Verwendung des juvenilen Slangs Yolo, das für ‚you only live once‘ steht, eine Form des Carpe diem). Durch den Titel des Beitrags „Get off the beach, Robin“, wird darauf verwiesen, dass sich die Erklärung Robins auf seine Anwesenheit am Strand bezieht (mit Blick auf das Datum ein Bezug auf den Spring Break, zu dem amerikanische Jugendliche die Strände Floridas für Feiern aufsuchen). Batman unterbricht Robin mit einer Ohrfeige und der Begründung, Robin (als „prick“ beleidigt) würde damit Alfred (den Butler Batmans als Repräsentant älterer Menschen) töten. Diese Szene wird außerhalb der Sprechblasen untertitelt damit, dass es nicht um einen selbst gehe. Darin wird der/die Betrachter*in adressiert und zu Rücksichtnahme aufgefordert. Im Kontext der Hashtags „coronavirus“ und „staythefuckhome“ wird verstehbar, dass es sich hierbei („It’s“) um Schutzmaßnahmen im Zuge der Pandemie handelt, v. a. um den Aufruf zu Hause zu bleiben (im Verweis auf den Titel „get off the beach, Robin“) und dass diese Maßnahmen zum Schutz älterer Menschen (repräsentiert durch Alfred und das Hashtag „alfredpennyworth“) dienen. Alfred zu töten meint, in dem Kontext von Titel und Hashtags, die Gefahr, sich während des Spring Breaks am Strand mit Corona zu infizieren und bei der Rückkehr andere anzustecken, die nicht jung und gesund genug sind, um die Infektion zu überstehen. Die in den komödiantischen Darstellungen isolierten Daheimbleibens beschriebenen Umstände werden hier durch kollektive Verantwortlichkeiten kontextualisiert (neben der Spannung zwischen Isolation und Kollektivität besteht die zweite Spannung darin, dass adäquates und angemessenes Handeln gerade im Verzicht auf Handlungsvollzüge besteht).

**Figure 11:**
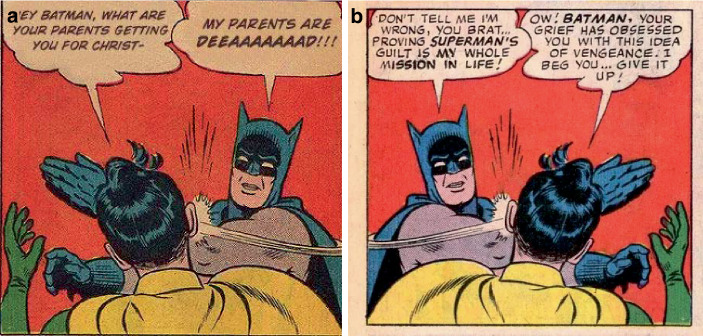

Das Batman-Slapping-Robin-Meme ist mit Blick auf seine Verwendung (Abb. 11a) und seine Vorlage (Abb. 11b) ein prototypisches Beispiel für die Transformation von Material in digitalen Räumen:

A. zunächst handelt es sich im Originalmaterial um eine *Modulation*. Die Comicvorlage stellt dabei eine Ausgabe von World’s Finest aus dem Jahr 1965 dar (Polygon 2020), in der Batman in einer alternativen Comic-Realität glaubt, Superman wäre verantwortlich für den Tod von Bruce Waynes Eltern. Es handelt sich also in Bezug auf die Comicwirklichkeit um eine fiktive Geschichte in der „realen“ Comicwelt, da es „normalerweise“ unvorstellbar ist, dass Batman Robin ohrfeigt. Dabei ist die Sprecherreihenfolge so, dass Batman spricht, Robin ihm widerspricht und Batman ihn daraufhin ohrfeigt.
B. handelt es sich im Memeformat um eine *Remediatisierung*, in der das Originalmaterial nicht im Sinne der Vorlage verwendet wird (im Gegensatz zu Abb. 1, 2, 3, 4 und 5). Eine der ersten Anwendungen als Meme (KnowYourMeme 2021b) stammt aus dem Jahr 2008 (Abb. 11a). Hier ist das Bild und damit die Sprecherreihenfolge gedreht: Robin fragt Batman, was dieser von seinen Eltern zu Weihnachten bekommen wird, wird aber durch Batmans Aussage, seine Eltern seien tot, und die Ohrfeige unterbrochen (im Original scheint Robin auszusprechen). Die Remediatisierung dient nicht der Darstellung, in der Robins Zweifel an Batmans Annahmen thematisiert werden. Vielmehr wird humoristisch der Mythos Batman und dessen Entstehungsgeschichte persifliert.
C. In der Verwendung in unserem Material handelt es sich dann um die Transformation im Rahmen des coronabezogenen Memes (Abb. 10a). Hier wird die humoristische Anwendung für eine ernste Meinung im Mantel satirischer Zuspitzung und im Verweis auf den Batman-Mythos transformiert (nämlich Alfred als älteren, fürsorglichen Butler und Teil der homosozialen männlichen Patchworkfamilie). Robin wird darin zum Repräsentanten einer (jugendlichen) Gruppe, die durch Batman mittels Gewalteinsatz „zur Vernunft“ gebracht werden soll. Der hinzugefügte Appell „It’s not about you!“, wie auch Titel und Hashtags zum Beitrag, unterstreichen dies (auch in der Forderung, zu Hause zu bleiben „staythefuckhome“ bzw. „shelterinplace“ als Verweis auf die Forderung, zu Hause Schutz zu suchen).^6^

**Figure 12:**
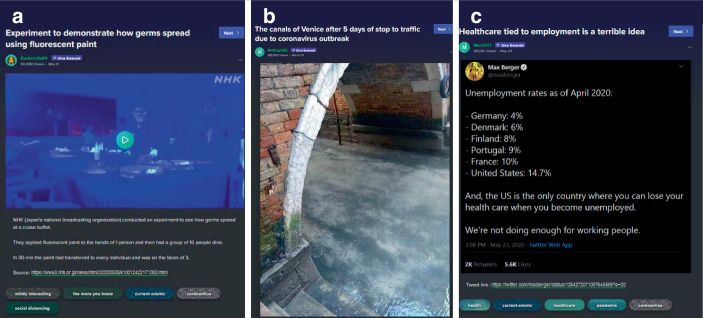

Neben kreativ-komödiantischen und Meinungsbeiträgen zeichnet den dritten Cluster ein höherer informativer Gehalt aus (38,6 % des Samples). Hierbei lassen sich drei weitere Subtypen rekonstruieren: aufklärende Beiträge (2,6 %; z. B. zu epidemiologischen Modellen und Ansteckungen; Abb. 12a), rein informative Beiträge (13,2 %; z. B. Beiträge zu leeren Straßen oder rückgehender Umweltverschmutzung; Abb. 12b) sowie kommentierte Informationen (14,3 %; also kommentierte Nachrichten, Statistiken u. Ä.; z. B. zu Arbeitslosenzahlen und Krankenversicherung; Abb. 12c). Wenige Beiträge referenzieren dabei Quellen – in Abb. 12c ist der angegebene Link z. B. lediglich ein Link zum Tweet. Teils werden Quellen in den Kommentaren eingefordert oder nachgeliefert und teils auch in den Beitrag eingebettet. Auch hier zeigt sich die Eigenheit der Plattform, Beiträge verschiedener sozialer Medien und aus verschiedensten Ländern (teils als persönliche Beiträge, teils als organisationale Medienbeiträge) zu filtern und zusammenzustellen.^7^

### Verhandelte Themen – zwischen Makro- und Mikroebene

**Figure 13:**
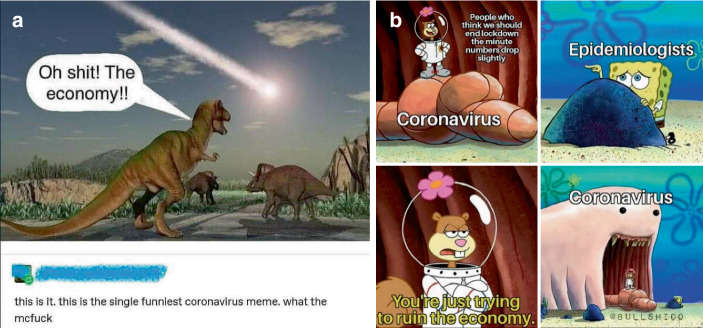

Neben den Medienformaten und den kommunikativen Mustern lassen sich thematische Differenzierungen der Beiträge rekonstruieren, die in vier Gruppen typisierbar sind. Ein großer Teil der Beiträge fokussiert auf Auswirkungen und den Umgang mit Corona in gesellschaftlichen Teilbereichen (gesamt 32,1 % des Samples; zusammengesetzt u. a. aus Beiträgen zu Wissenschaft, Wirtschaft, Medien und Politik). Dabei wird die Wissenschaft eher positiv besetzt. Demgegenüber überwiegen in der Verhandlung von Wirtschaft, Politik und Medien kritische Stimmen (als Meinungen oder kommentierte Informationen; Abb. 12c). Seitens der Wirtschaft werden Unternehmen gelobt, die sich an der Produktion von Schutzausrüstung beteiligen (Abb. 7b), kritisiert wird dagegen der Ruf nach finanzieller Unterstützung durch die Politik (als „bailout“; Abb. 7a), die Nutzung der Pandemie zu Werbezwecken (Abb. 10b) und der unzureichende Schutz der Arbeitnehmer*innen bzw. eine Kritik am Primat des Ökonomischen in Zeiten einer globalen Pandemie (Abb. 13). Medien, v. a. amerikanische Medienorganisationen werden hinsichtlich Falschinformationen und falscher Relevanzen kritisiert, auch finden sich oft in Kommentaren oder Titeln zu Informationen aus der ausländischen Presse kritische Bemerkungen, wobei die Plattform in ihrer katalysatorischen Filterfunktion als eine Art Gegenmedium dargestellt wird, z. B. in der Idee, ein Post auf der Plattform könne dazu führen, dass Medienorganisationen Nachrichten aufgreifen (als Untertitel zu einem Beitrag zu Protesten z. B. „Signal boost these people. The media should focus on these people.“; Abb. 16b).

**Figure 14:**
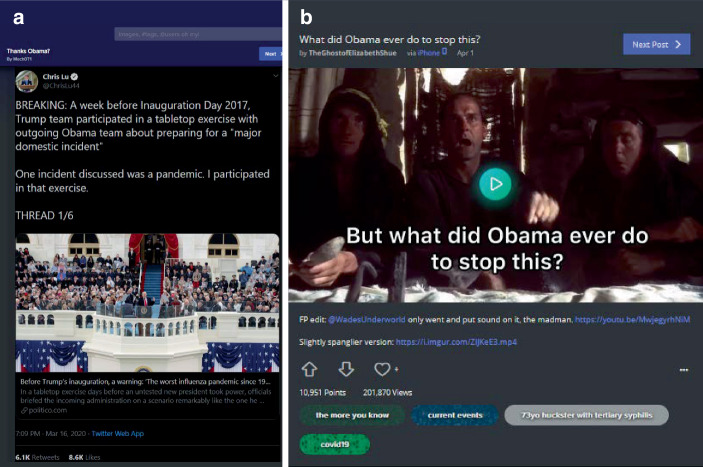

Unter den Teilbereichen überwiegen Beiträge zur Politik (23,7 % des Gesamtsamples; Abb. 14 und 8b; Abb. 12c; Abb. 13b), wobei Kritik an Donald Trump und der Politik der Vereinigten Staaten (Abb. 14) im Vergleich zu anderen Nationen überwiegt (Deutschland und Neuseeland werden z. B. als positive Beispiele behandelt).

Hier zeigt sich, dass Soziale Medien nicht nur unterhaltsame Beiträge und persönliche Erzählungen verhandeln, sondern dass sie auch als meinungsbildende und -stabilisierende Plattformen dienen (selten in Referenz der Quellen). Hier zeigen sich besonders plattformeigene thematische und ästhetische Differenzen der Verhandlung der Pandemie, z. B. zu stärker eigenen, persönlichen und kreativen (und weniger politischen und informativen) Beiträgen zu Corona auf Instagram^8^ oder zu mehr textbasierten, medienreferenzierenden und eigenen Meinungsbeiträgen auf Twitter.

**Figure 15:**
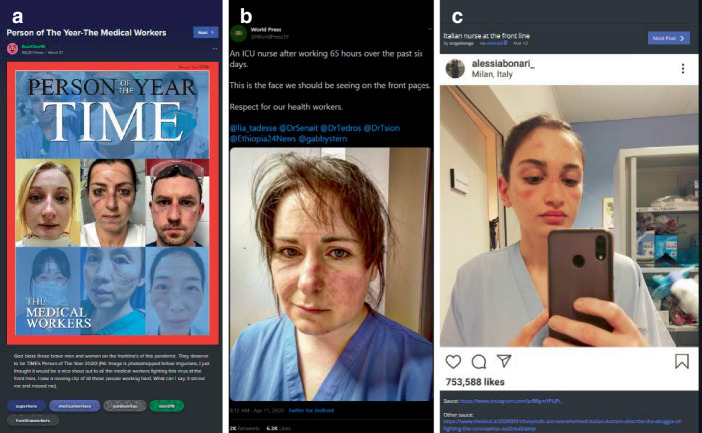

Eine zweite Themengruppe verhandelt die Pandemie hinsichtlich der konkreten Folgen, der zu ergreifenden Schutzmaßnahmen, daran orientierter Ratschläge, aber auch hinsichtlich des als Held*innen verstandenen medizinischen Personals (27,6 % der Beiträge). Hinsichtlich der Held*innen ist auffällig, dass v. a. weibliches Pflegepersonal besonders viel Zuspruch erhält (Abb. 15). Es finden sich aber auch Ratschläge zum Händewaschen, zur Herstellung von eigenem Desinfektionsmittel oder aber der Vorbereitung im Falle einer Infektion (z. B. zur Hausapotheke als Ratschlag einer selbstbezeichneten „registered nurse“, also einer Fachkraft).

**Figure 16:**
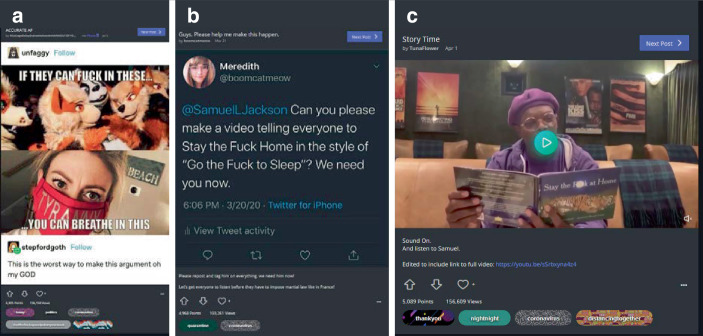

Es ist die Eigenheit der Vielfalt der Beiträge, dass solche, die ernst, authentisch, informativ sind, neben humoristischen, satirischen Posts stehen. So finden sich z. B. zu den Schutzmaßnahmen (Maskentragen und Social Distancing), neben dokumentarischen Bildern, auch unterhaltsame Darstellungen (Abb. 16). Hier zeigen sich Wechselwirkungseffekte zwischen Sozialen Medien, Medienorganisationen und Personen. So findet sich ein Tweet vom 20. März 2020, der tags darauf auf Imgur verbreitet wurde, der Schauspieler Samuel Jackson müsse im Stil einer Einschlafgeschichte auf die Notwendigkeit des Zuhausebleibens hinweisen. Am 1. April 2020 folgt darauf ein Beitrag mit einem Videoausschnitt und Link zu einem YouTube-Video von Jimmy Kimmel Live (einer Late-Night-Show aus den USA), in dem Samuel Jackson auf einer Couch sitzt und aus einem vermeintlichen Buch mit dem Titel „Stay the F*ck at Home“ vorliest (in Verweis auf das Einschlafbuch „Go the F*ck to Sleep“ von Adam Mansbach, das Samuel Jacksons als Audiobuch eingesprochen hat).^9^ Jackson verweist im YouTube-Video darauf, dass Adam Mansbach auf ihn zugekommen sei; im Kontext der Beiträge auf der Plattform erscheint dies aber (auch) als Leistung der Vernetzung und Reichweite der eigenen Beiträge.^10^

**Figure 17:**
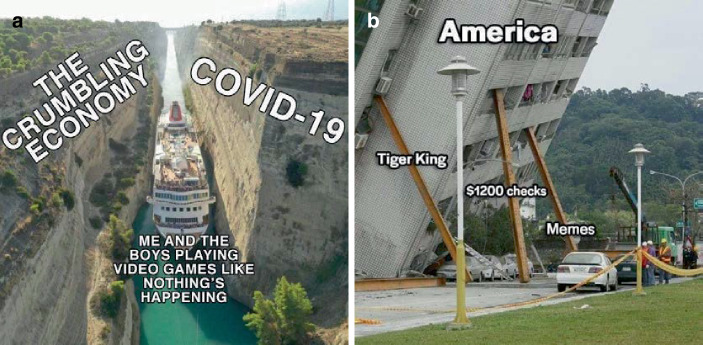

Die dritte Themengruppe behandelt den Umgang mit der Pandemie, v. a. den persönlichen Folgen des Zurückgeworfenseins auf den eigenen Haushalt (20,9 % im Sample; Abb. 9). Hier finden sich vorrangig kreativ-komödiantische Beiträge, die einen dramatisierten Einblick in grundsätzliche Differenzen zum vorpandemischen Alltag markieren, dabei auf Probleme der Vereinbarkeit von Homeoffice und Kinderbetreuung oder auf Videokonferenzprobleme verweisen^11^ oder sie handeln von hedonistischen Copingstrategien, wie etwa Alkoholkonsum, Online-Shopping und Masturbation. Dabei finden sich auch Verweise auf eine Einordnung der Pandemie in einen gesellschaftlichen und historischen Kontext als außergewöhnliche Situation. Dem werden dann (satirisch) die vergleichsweise profanen Strategien gegenübergestellt (Computerspielen, TV-Serien wie Tiger King schauen, aber auch Memes; Abb. 17), welche im Umgang helfen (das Schiff durch den engen Kanal zu manövrieren; Abb. 17a) bzw. die Gesellschaft stabilisieren (Abb. 17b).

**Figure 18:**
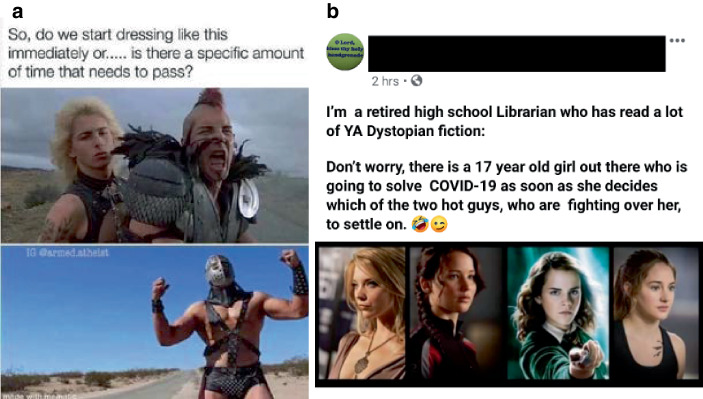

**Figure 19:**
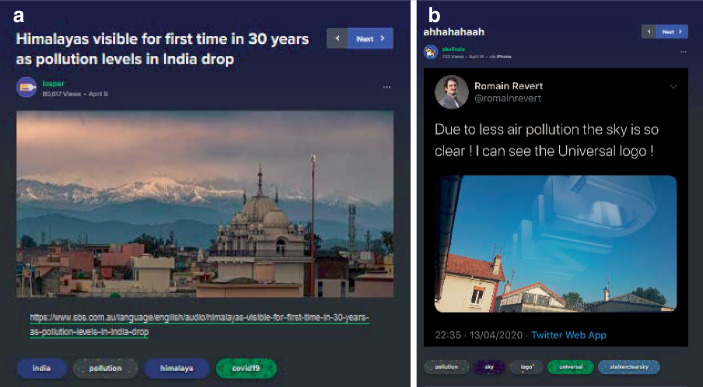

Oft finden sich bei der Standortbestimmung Verweise auf popkulturelle Referenzen, v. a. dystopisch-apokalyptische Filme wie Mad Max (Abb. 18a) oder der Verweis auf bestimmte Filmtropen von Heldinnen in großen Konflikten (Abb. 18b), aber auch TV-Serien wie Black Mirror werden als filmische „Paten“ einer dramatischen Gegenwartsbeschreibung genutzt. So finden sich auch Modulationen des Themas saubere Städte infolge verminderten Verkehrs (Abb. 12b). In der Modulation wird dies satirisch aufgegriffen und mit der popkulturellen Selbstverortung so zusammengeführt (Abb. 19), dass es erscheint, als befinde sich die Person wie „in einem Film“, deutlich erkennbar daran, dass das im Himmel sichtbare/eingefügte Logo der Produktionsfirma (hier: Universal) von hinten zu sehen ist, also für einen Beobachter außerhalb der Atmosphäre lesbar erscheint.

**Figure 20:**
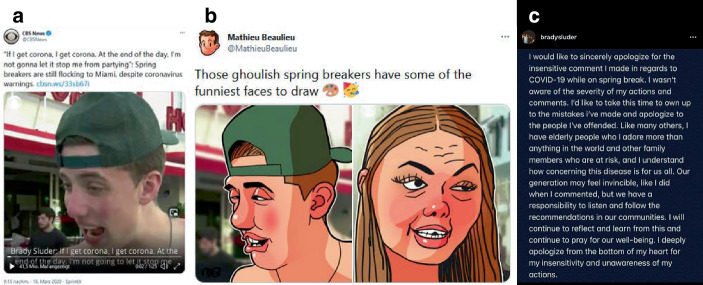

Die vierte Gruppe befasst sich mit persönlichem Fehlverhalten, Gegnern von Schutzmaßnahmen, Corona-Leugnern (als ‚COVIDiots‘ gelabelt) und Verschwörungstheorien (16,1 % des Samples). Hier zeigt sich in der Abgrenzung nach außen (auch in Kritik an politischen/wirtschaftlichen Organisationen und für diese handelnden Personen) eine Markierung geteilter Einstellungen, die Pandemie und die damit verbundene Bedrohung ernst zu nehmen (auch in unernsten, unterhaltsamen Beiträgen), die Schutzmaßnahmen zu befürworten und dahingehend unverantwortliches Verhalten zu verurteilen (Abb. 13b). So werden Proteste zur Wiederöffnung stark kritisiert (z. B. durch die Abbildung von als lächerlich/beschämend bezeichneten Forderungen auf Demonstrationsschildern, zum Friseur gehen zu wollen). Auch unverantwortlich erachtetes, aber nicht verbotenes Verhalten, wie Flugreisen und Urlaub (Abb. 1 und 10a) werden auf der Plattform geächtet (bzw. solche Beiträge erhalten eher Zuspruch). So findet sich häufig der Verweis auf ein Video von CBS News (Abb. 20a; am 20. März auf Twitter gepostet), in dem betrunkene Jugendliche zum Spring Break interviewt werden („If I get corona, I get corona. At the end of the day, I’m not gonna let it stop me from partying“). Dieses Video wurde zum Ausgangsmaterial für Karikaturen, die Mathieu Beaulieu am selben Tag auf Twitter postete, in denen die Anzeichen der Trunkenheit überzeichnet werden (Abb. 20b). Diese Bilder werden auch auf Imgur geteilt und finden sich als Kommentarbilder wieder. Das Video, wie die Karikaturen, verbreitete sich und führte dazu, dass sich die Personen entschuldigten (so die in Abb. 20 abgebildete Person auf ihrem Instagramprofil), was wiederum auf Imgur als Beitrag geteilt wurde. Dies ist bemerkenswert, nicht nur hinsichtlich der Remediatisierung eines Video-Nachrichtenbeitrags einer Medienorganisation in eine Bild-Karikatur eines Twitternutzers und von dort in ein Kommentierungsbild für Imgur, sondern auch hinsichtlich eines Kaskadeneffekts, der die betroffenen Personen erheblich unter Erklärungsdruck brachte.

**Figure 21:**
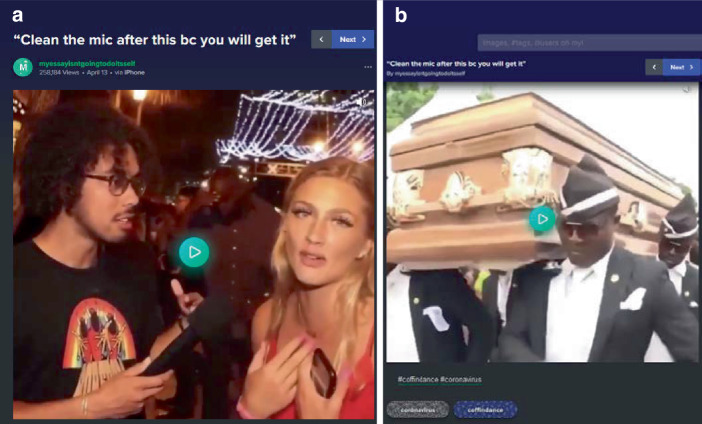

Eine coronaeigene Form der Kommentierung und das populärste Meme im Material sind die tanzenden Sargträger (Coffin Dance Meme; vgl. KnowYourMeme 2021c). Die Originalvorlage geht dabei auf ein Video von BBC News Africa auf Twitter von 2017 zurück, in dem tanzende ghanaische Sargträger portraitiert wurden. Dieser Ritus wurde mutmaßlich im Februar 2020 zunächst im Rahmen sogenannter Fail-Videos (als Spielart der Schadenfreude) mit einem EDM-Musiktrack gepaart, um scheiternde und halsbrecherische Aktionen zu kommentieren. Im Zuge der Coronapandemie wird der Coffin Dance zur Kommentierung persönlichen Fehlverhaltens von Privatpersonen und Politiker*innen genutzt (vgl. Abb. 21). Im Stil der Failclips z. B. in einem Beitrag, der einen Journalisten zeigt, der eine Frau interviewt, die am Ende meint, sie habe Corona und er solle das Mikrophon danach säubern, sonst würde er es auch kriegen. Der Interviewer wirkt sprachlos und schaut in die Kamera, dazu setzt die elektronische Musik ein und Szenen des BBC-Clips der tanzenden Sargträger folgen (im selben Stile findet sich z. B. der Zusammenschnitt einer Pressekonferenz Donald Trumps mit dem Coffin Dance). Damit wird die Bedrohung einer Infektion und eines potenziell tödlichen Verlaufs durch den Verweis auf eine Beerdigung dargestellt und in Anlehnung an die Failclips und in Kombination mit der Musik und einer – für andere Menschen – unvertraut und befremdlich wirkenden Praxis die damit verbundene Kritik mit einem gewissen Unterhaltungswert präsentiert. In anderen Varianten werden vor dem Fehlverhalten Szenen gezeigt, welche die Sargträger ohne Sarg wartend und wie für eine Gruppenaufnahme aufgestellt zeigen, womit der Coffin Dance das so kommentierte Video rahmt (im Sinne einer drohenden Gefahr und einer dann folgenden Konsequenz).

**Figure 22:**
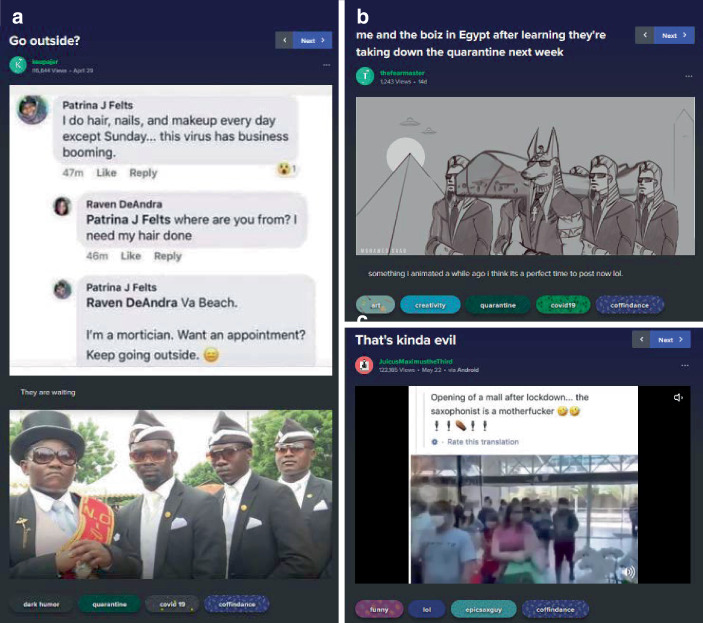

**Figure 23:**
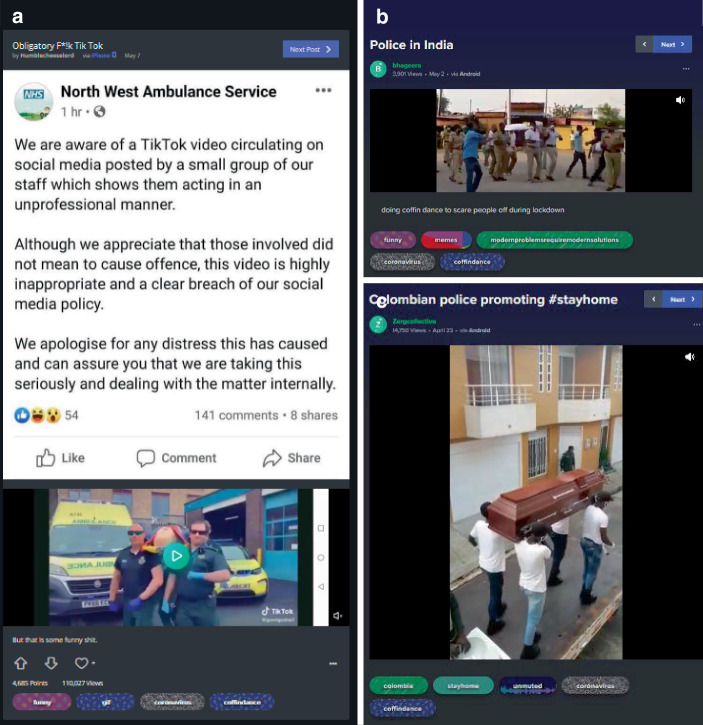

Das Meme ist besonders interessant, da es verschiedene (auch mediale) Abwandlungen erfährt (z. B. als Bildkommentar in Darstellung der wartenden Sargträger, als gezeichnete Comic- und animierte Cartoonfiguren). Es finden sich aber auch Verweise, dass das Meme in realweltlichen Situationen genutzt wird, z. B. als Straßenreklame oder als gespielte Melodie auf einem Saxophon bei der Wiedereröffnung eines Einkaufszentrums (Abb. 22). Es wird auch von Polizeikräften aufgeführt, z. B. bei öffentlichen Kundgebungen, welche erlassene Schutzmaßnahmen verkünden und deren Einhaltung fordern (Abb. 23). So wird in Beiträgen auf Peru, Kolumbien, Großbritannien und Indien verweisen, in denen dies vorgekommen sein soll. Die Popularität des Memes im Kontext der Pandemie wird dann von der, in der BBC-Dokumentation vorgestellten, Gruppe aufgenommen und u. a. in einem nachfolgenden Beitrag von BBC News Africa vom Mai 2020 und in anderen Fernsehbeiträgen thematisiert. So führen die Sargtänzer den Tanz aus dem Ursprungsvideo mit Masken auf, fordern das Tragen von Masken und drohen, sonst mit den Personen „zu tanzen“.

## Fazit: plattformbezogene Kommunikationsmodi und alltagspraktische Copingstrategien

### Reiz plattformspezifischer Umgangsformate – Mediensoziologisches Fazit zu Sozialen Medien und Memes

**Figure 24:**
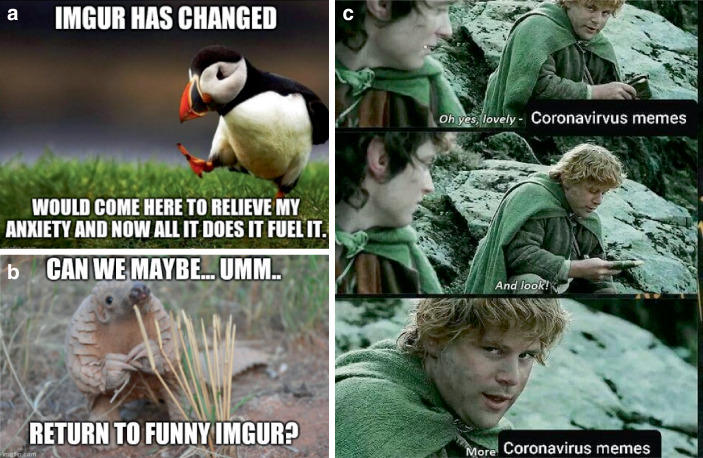

Im Rahmen der Verbreitung von coronabezogenen Beiträgen finden sich auch Beiträge, die diese Entwicklung kritisch sehen und eine Veränderung der Plattform markieren. Vor allem geht es dabei um eine wahrgenommene Abnahme ablenkender, humoristischer Beiträge, die Zunahme von politischen Beiträgen und eine größere Verunsicherung. Diese Reflektion wird u. a. über Memes formuliert (Abb. 24) und dabei als unpopuläre Meinung bzw. als vorsichtige Bitte durch die Verwendung der jeweiligen Vorlage markiert. Auch wird eine Übersättigung mit Corona-Memes verhandelt, z. B. durch Zitation einer Szene aus Herr der Ringe, in der Samwise sich über den eintönigen Proviant beschwert (als sarkastische Formulierung „Oh yes lovely Coronavirus Memes“/„And look“/„More Coronavirus Memes“).

Seitens der User*innen wird eine Transformation der Plattform-Inhalte in der Adaption der plattformeigenen Kommunikationsweisen deutlich. Nicht nur, dass sich die Plattform hinsichtlich der Inhalte wandelt, sondern auch, dass unterhaltsame Kommunikationsformen (Memes) stärker zur themenbezogenen Meinungsartikulation genutzt werden. Aber auch hinsichtlich der begrifflichen Fassung von Memes zeigen sich Herausforderungen, die besonders am populären Coffin Dance deutlich werden. Dieses Meme geht über die Verhandlung von Bild-Text-Relationen hinaus und schlägt sich in verschiedenen digitalen und realweltlichen Kontexten nieder.

Neben diesen Transformationen von Plattform und Memes zeigen sich im Material typische Aspekte Sozialer-Medien-Plattformen. So zeigen sich im kursorischen Vergleich zu anderen Plattformen (Instagram, Reddit u. a.) Aspekte einer plattformeigenen Umgangssprache (als *platform vernacular* am Beispiel von Instagram Gibbs et al. 2014). So handelt es sich weniger um reine Textbeiträge (wie auf Twitter), sondern stärker um eine bildbasierte Kommunikation, die aber im Vergleich zu Instagram nicht über die Präsentation des eigenen Selbst/Körpers und selbstgemachter Fotos/Videos funktioniert. Gegenüber Twitter und Instagram handelt es sich um eine anonymere und – über die Frontpage – zentralisiertere Kommunikation, die auf eine Reihe von Vorlagenmaterial (Medienbeiträge, Popkulturmaterial, Memes etc.) zurückgreift, aber auch eigens erstellten Inhalt anerkennt. D. h., die – Sozialen Medien zugeschriebene – kreative Produktivität der Nutzer*innen (als *vernacular creativity*; Burgess 2007) ist sowohl durch plattformeigene technische Restriktionen als auch durch soziale Konventionen geprägt. Dementsprechend zeigen sich hier Anzeichen einer plattformeigenen und spezifischen Kreativität (*platform vernacular creativity*) als Zeichen einer digitalen partizipativen Kulturformation (*participatory culture*; Fuchs 2014). Wobei gegenüber dieser Engführung auf (eigen-)kreative Beiträge hinsichtlich der hier beschriebenen Plattform verdeckt wird, wie viel Weiterverwertung von Beiträgen anderer Plattformen stattfindet. Neben der Beförderung eigenkreativer Beiträge leisten Plattformen zudem eine Art Fremdbeobachtung und Auswahl von Beiträgen anderer Plattformen nach eigenen Bewertungslogiken. Es ließe sich hier komplementär von plattformspezifischen Filterschleifen (*platform vernacular filterloops*) sprechen.

Plattformeigen scheinen in unserem Fall nicht nur die Formen der Kommunikation, sondern auch die vermittelten Einstellungen. Über das Material hinweg zeigen sich als anerkannte und geteilte Beiträge v. a. solche, die sich gegen Corona-Leugner, Verschwörungstheoretiker stellen, solche die die Pandemie und deren Gefahren ernstnehmen, Schutzmaßnahmen befürworten und wirtschaftliche, wie auch politische Entscheidungen kritisieren, welche die Eindämmung und Folgen der Pandemie nicht ernst nehmen. Somit scheint die hier behandelte Plattform in den populären Beiträgen, vor allem in der Kritik an Einzelpersonen und Organisationen einig und dabei durchaus als plattformspezifische „Filter Bubble“ (Pariser 2011; Davies 2018) beschreibbar. Verstärkt wird dies durch die Weitergabe größtenteils unbelegter Informationen, welche v. a. hinsichtlich der darin deutlichen geteilten Einstellung Anerkennung finden. Dies zeigt u. E. nach auch an, dass eine stärker plattformvergleichende Forschung die Varianz sozialer Medien (in Inhalten, Formen und Einstellungen) gegenüber der allgemeinen Verhandlung dieser stärker in den Blick nehmen sollte (Vicari und Murru 2020, S. 3).

An der Beitragsvielfalt zeigt sich, dass eine Fokussierung auf Beiträge als Teile eines politischen Diskurses, als Unterhaltungsform (Dynel 2020; Saint Laurent et al. 2021) oder auf bestimmte Länder (Hussein und Aljamili 2020) unzureichend scheinen, sprengt das hier beschriebene Material doch diese Eingrenzungen. Vielmehr scheint der Reiz der Plattform gerade in der Hybridität des Materials zu bestehen (Luchman et al. 2014): selbsterstellte komödiantische Videos, eine Art kuratierte Auswahl bester Tweets, Medienbeiträge aus anderen Ländern, zudem (seltener) recherchierte und umfangreiche Analysen, für die Nutzer*innen teilbare Meinungen und Einstellungen als Form der Selbstvergewisserung und Gruppenbildung (DeCook 2018). In schneller und selbstbestimmter Folge lassen sich so verschiedene Mediennutzungsmotive verbinden – Unterhaltung, Information, Meinungsbildung, sozialer Austausch und Zusammenschluss – und es lässt sich über Likes, Kommentare und eigene Beiträge einfach von der Rezipient*innen- zur Produzent*innenperspektive wechseln.

### Verständnis und Copingstrategien von Alltagsakteuren in Krisenzeiten

**Figure 25:**
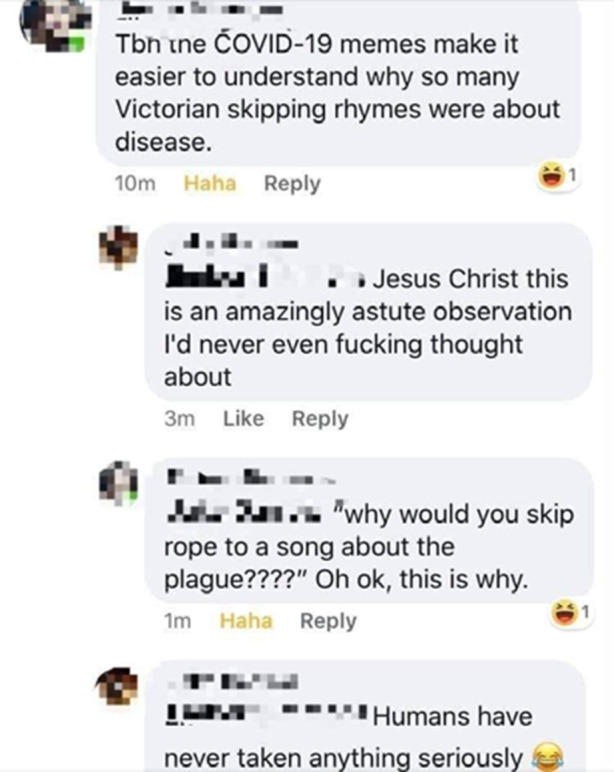

Bezüglich der Verhandlung der Pandemie als Krise zeigt sich im Material – durch die plattformeigene popkulturell-referenziell angereicherte Bildsprache – durchweg die Thematisierung einer außergewöhnlichen, gleichsam „filmreifen“ und darin besonderen Situation. Dies auf Individual- wie auch gesellschaftlicher Ebene. Entsprechend dieser Krisenerfahrung sind auch die persönlichen Umgangsweisen in zweierlei Hinsicht besonders: Zum einen handelt es sich um die Darstellung der Extensivierung von Handlungen (Online-Shopping, Genussmittel etc.), zum anderen um neue Handlungsmuster bezüglich der Schutzmaßnahmen, die oft humoristisch dokumentiert werden und neben vorrangig informativen und meinungsbildenden Beiträgen stehen. Es mag der Verdacht naheliegen, dass die hier beschriebenen Phänomene auf eine Besonderheit digitaler und gegenwartsgesellschaftlicher Kontexte hinweisen. Vielmehr scheint es aber in einer Linie volkstümlicher Verhandlungen (so auch eine Lesart des Englischen *vernacular*) historischer Ereignisse in verkürzenden, unterhaltsamen Medienformaten zu sein, bedenkt man z. B. Kinderreime und Lieder zu grausigen und darin besonders populären Kriminalfällen, wie von Fritz Haarmann^12^ in Hannover 1924 oder Lizzie Borden^13^ in Massachusetts 1892. Hier scheint die Verbindung einer realen und bedrohlichen Situation mit unterhaltsamen Melodien oder kindlichen Spielen eine Form der Unterhaltung und des Umgangs, aber auch eine Form volkstümlicher Geschichtsschreibung und Überlieferung zu sein. Wie die unreferenzierten Beiträge in den Sozialen Medien beinhalten auch diese volkstümlichen Erzählungen eine Reihe an Fehlern (z. B. zur Adresse Fritz Haarmanns oder der Anzahl an Axthieben bei Borden). Andererseits dienen die Eingängigkeit der Reime, die Dramatik des Inhalts und der Rückgriff z. B. auf eine einschlägige Melodie der schnellen Wiedererkennbarkeit und Verbreitung der Informationen – und dies derart erfolgreich, dass diese Reime und die damit verbundenen historischen Ereignisse als eine Art Alltagswissen bis heute präsent bleiben. Die einfache Erstellung und Verbreitung von Inhalten in Sozialen Medien, die Verweise auf bekannte populärkulturelle Referenzen sowie deren Nutzung als Vorlage zur Modifikation und die Kombination aus dramatischen Ereignissen und unterhaltsamer Darstellung ist vergleichbar den volkstümlichen Reimen eine Art „Erfolgsrezept“ für Alltagsakteure, an der gesellschaftlichen Verhandlung solcher Ereignisse teilzuhaben. Spannenderweise findet sich diese Reflektion auch im Material selbst (Abb. 25; wobei auch hier wieder die Problematik der fehlerhaften Argumentation insofern greift, als dass der Bezug von Abzählreimen zur mittelalterlichen Pestpandemie dem Forschungsstand nach nicht haltbar ist; Opie und Opie 1997, S. 364 f.).

## Footnotes

1 Auch ohne Benutzerprofil können Beiträge betrachtet werden, das Posten/Kommentieren von Beiträgen ist mit Profil möglich (wobei Pseudonyme verwendet werden, was Imgur zufolge zu authentischeren Aussagen führt; Imgur 2021). Die Nutzer*innen sind überwiegend gebildet, männlich, weiß und junge Erwachsene (Imgur 2017).

2 Zur Erstellung des Artikels (Januar 2021) umfasst das Hashtag 35.000 Postings. Trotz geringerem Zuwachs lassen sich immer noch coronabezogene Beiträge finden, die allerdings kaum mit coronabezogenen Hashtags versehen sind.

3 Mit dem Zugang sind auch Limitationen verbunden: So ist die plattformspezifische User*innengruppe hinsichtlich der Nationen, die sie gewissermaßen vertreten, divers; demografisch scheinen die Nutzer*innen dagegen homogen und damit nur einen Ausschnitt der Bevölkerung zu repräsentieren.

4 Teils wurden Beiträge mehrfach kodiert; die folgenden Prozentangaben beziehen sich immer auf die primäre Kodierung hinsichtlich der Kategorieausprägung.

5 Dies kann aus der Unübersichtlichkeit z. B. von Twitter herrühren, womit auf Imgur Beiträge vorkommen, die im eigenen Twitterfeed nicht angezeigt werden. So erweitert die Imgur-Filter(blase) die Twitter-Auswahl. Die Weiterleitung dient mutmaßlich nicht nur der Generierung von Aufmerksamkeit mittels eines erfolgreichen und erprobten Beitrags, sondern auch als Katalysator für die Weiterverbreitung.

6 Ein Gegenstück dazu sind Beiträge, die das verantwortungslose Verhalten älterer Menschen thematisieren. So z. B. in einem Video betitelt als „Millennials right now“, in dem ein älterer, weißhaariger Vater wie ein trotziger Teenager auftritt und fordert, für „beer with the guys“ rauszudürfen („Jason led his dad go“), während der etwa Ende 20-jährige Sohn ihm dies verbietet („Well, I am not Jason“), woraufhin der Vater trotzig erwidert „you never let me have any fun“.

7 Neben kreativ-komödiantischen, informativ-kommentierenden und Meinungsbeiträgen gibt es noch einen kleinen Teil von Erzählungen (9,3 % im Sample und 4,8 % sonstige Beiträge; Abb. 8c).

8 So zumindest im Vergleich zu einer unveröffentlichten Arbeit von Michael R. Müller zu Corona auf Instagram, die wir dankenswerterweise einsehen konnten.

9 Der Inhalt ist der reimenden Art von Einschlaftexten für Kleinkinder nachempfunden: „Motherf**ker it’s no time to gamble, look around, you’re not at a casino. Just stay the f**k home as if your name is Trenton Quarantino. Sure, you can still see your friends. Use the motherf**king app on your phone. But unless you just ran out of groceries, please! Stay the f**k at home!“.

10 Auf dem Twitterkanal der Posterin ist der Tweet an den Autor/dessen Twitteraccount gerichtet. Es folgen Kommentare, die auch den Autor und Jackson adressieren, der Post sei auf Facebook und Reddit mit großem Zuspruch geteilt worden.

11 So zeigt ein Beitrag mit dem Titel „Working from Home during Quarantine“ eine Frau mit dem Rücken zur Kamera an einem Tisch mit Laptop, während hinter ihr auf dem Boden drei Kinder geknebelt und gefesselt liegen. Ein anderer Beitrag „Shoutout to all the new day-drinking parents:“ zeigt ein eher dokumentarisches Foto (aus dem Inneren einer Wohnung nach draußen), auf dem eine Frau mit einem Weinglas in der Hand, in Pyjamas nachmittags mit einem fahrradfahrenden Kind den Gehweg entlanggeht. Der Kommentar dazu ist, dass dies *das* Bild sei, das die Quarantäne 2020 zeigt (gemeint scheint der Lockdown).

12 „Warte, warte nur ein Weilchen, bald kommt Haarmann auch zu dir, mit dem kleinen Hackebeilchen macht er Schabefleisch aus dir.“ Eine Remediation der Melodie und in Anlehnung an den Text des damals populären Liedes „Warte, warte nur ein Weilchen, dann kommt auch das Glück zu dir“ von Walter und Willi Kollo aus der Operette Marietta von 1923. Das Haarmannlied folgte 1924 (Gules 2014).

13 „Lizzie Borden took an axe/And gave her mother forty whacks./When she saw what she had done,/She gave her father forty-one“ (Burton 2012).
